# Tyrosinemia type 1: a rare and forgotten cause of reversible hypertrophic cardiomyopathy in infancy

**DOI:** 10.1186/1756-0500-6-362

**Published:** 2013-09-09

**Authors:** Sarar Mohamed, Mohammed A Kambal, Nasir A Al Jurayyan, Abdulrahman Al-Nemri, Amir Babiker, Rana Hasanato, Abdullah S Al-Jarallah

**Affiliations:** 1 Department of Pediatrics, College of Medicine, King Saud University, Riyadh 31461, P.O. Box 2925, Saudi Arabia; 2 Department of Clinical Chemistry, College of Medicine, King Saud University, Riyadh, Saudi Arabia; 3 Cardiac sciences Department, College of Medicine, King Saud University, Riyadh, Saudi Arabia

**Keywords:** Tyrosinemia type 1, Cardiomyopathy, 2-Nitro-4-trifluoromethylbenzyl 1, 3 cyclohexanedione, Infant

## Abstract

**Background:**

Tyrosinemia type 1 (TT1) is an autosomal recessive disorder caused by deficiency of the enzyme fumarylacetoacetate hydrolase (FAH). TT1 usually presents in infancy with features suggestive of liver disease or with sepsis-like symptoms.

**Case presentation:**

We report two Saudi siblings with TT1. Case 1 was a male infant who presented at 2 months old with fever, vomiting and refusal of feeding. Examination revealed a sick-looking infant with signs of severe dehydration and hypovolemic shock. He was jaundiced, and had hepatomegaly and elevated liver enzymes. Echocardiography was performed in light of a lack of response to inotropes, and revealed biventricular and interventricular septal hypertrophies. The ventricular ejection fraction was 65%. Urine organic acid analysis showed elevated succinylacetone, consistent with a diagnosis of TT1. An FAH gene study identified a c.1 A > G homozygous mutation. This patient responded well to intensive cardiorespiratory therapy, tyrosine-free formula, and oral 2-nitro-4- trifluoromethylbenzyl 1, 3 cyclohexanedione (NTBC). Echocardiographic findings reverted to normal after 4 weeks. Case 2 was the younger brother of Case 1, and was born 6 months after his brother had been confirmed with tyrosinemia. Pregnancy and delivery were uneventful. Serum amino acid and organic acid analyses 4 days after birth confirmed tyrosinemia. DNA analysis identified a c.1 A > G homozygous mutation, as in his brother. Echocardiography was normal. Special formula and NTBC were commenced on day 7 of life. The infant remained asymptomatic after 9 months of follow-up.

**Conclusions:**

These cases highlight TT1 as a treatable cause of cardiomyopathy in children. It also supports the idea that early diagnosis and treatment may prevent the development of cardiomyopathy associated with tyrosinemia.

## Background

Tyrosinemia type 1(TT1) is an autosomal recessive disorder caused by deficiency of the enzyme fumarylacetoacetate hydrolase (FAH), which catabolizes fumyralacetoacetate into fumarate and acetoacetate [1]. The pathophysiology of the disease is explained by the subsequent accumulation of tyrosine and its metabolite succinylacetone in the liver, kidney and peripheral nerves, leading to dysfunction of these organs [1,2]. TT1 usually presents in early infancy with failure to thrive, vomiting, jaundice, hepatomegaly, elevated liver enzymes and bleeding tendency [3]. Late clinical features of TT1 include peripheral neuropathy, Fanconi-like syndrome and vitamin D-resistant rickets [1]. Cardiomyopathy is a rare condition in childhood [4], and the Pediatric Cardiomyopathy Registry (PCMR) estimated the incidence of childhood cardiomyopathy in the United States to be 1.13 cases per 100,000 children [4]. Cardiomyopathy may be the predominant finding in a number of inborn errors of metabolism, including mitochondrial disorders, glycogen storage disorders, carnitine transporter defects, fatty acid oxidation defects and lysosomal storage diseases [4,5], and some studies have reported cardiomyopathy as an unusual and rare manifestation of TT1 [6-9]. Herein we report two Saudi brothers with TT1. The older sibling presented with hypertrophic cardiomyopathy but responded well to medical treatment, while the second infant remained asymptomatic after being diagnosed by selective screening.

## Case presentation

**Figure 1:**
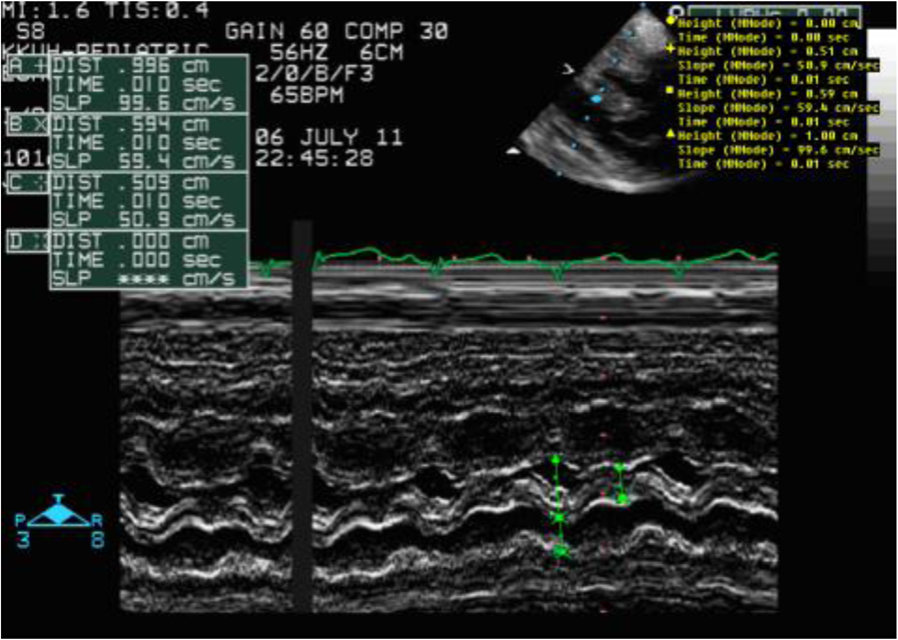
M mode measurements revealing an interventricular septum size of 0.59 cm before treatment.

**Figure 2:**
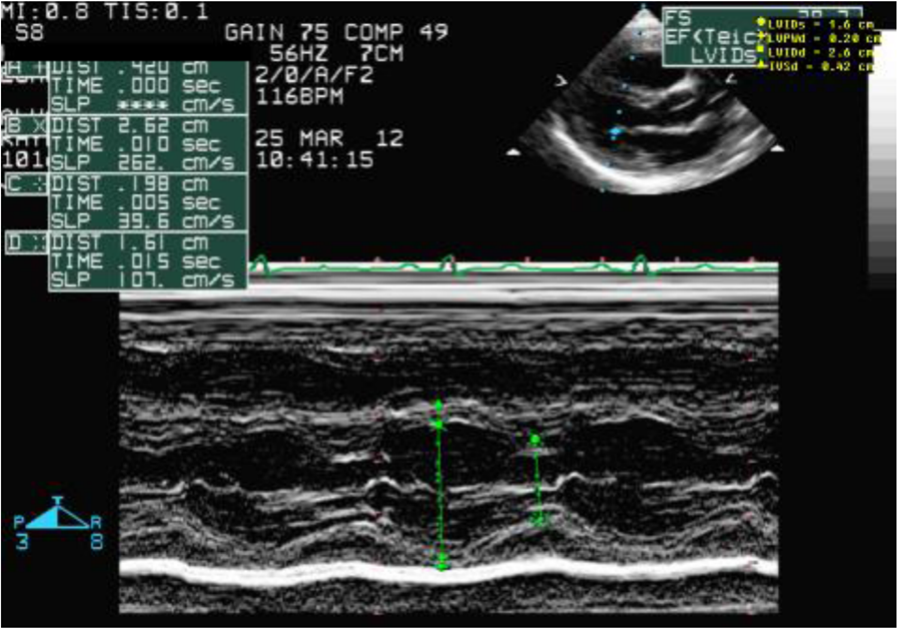
M mode measurements revealing an interventricular septum size of 0.42 cm after treatment.

**Figure 3:**
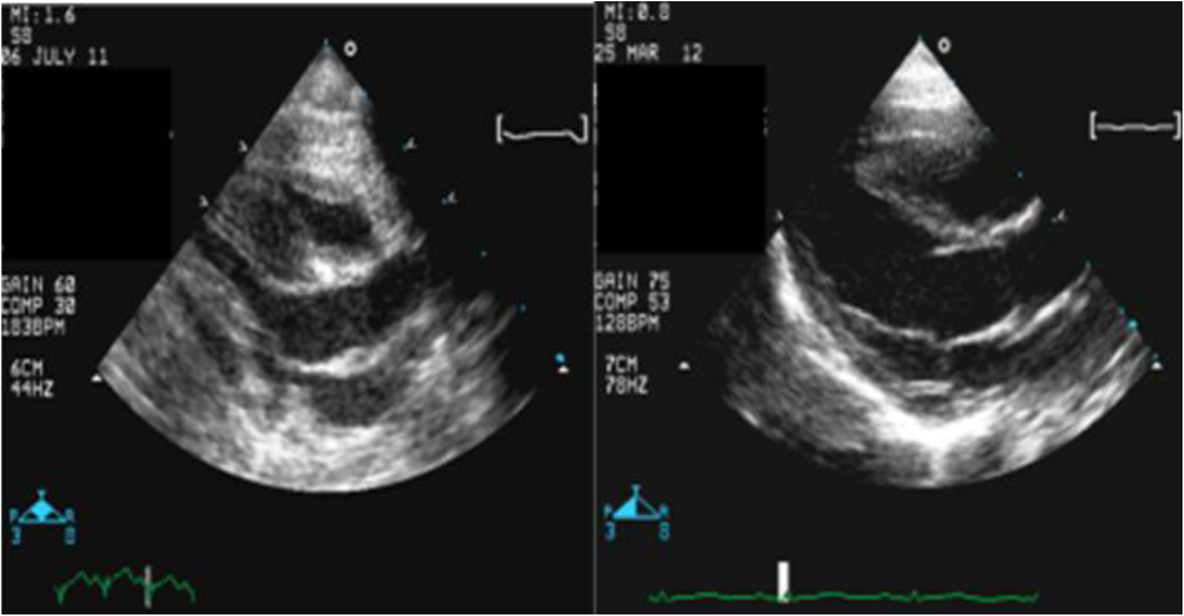
Long axis view revealing improvement in interventricular septum thickness with treatment.

Case 1 was a previously well, 2-month-old Saudi boy born to first cousin parents. The couple had two other children, one of whom had died in early infancy with an unexplained acute febrile illness. The patient was born following an uneventful pregnancy and delivery. He presented with a 2-day history of fever, poor feeding, difficulty in breathing and lethargy. His weight, length and head circumference were < 5th centile. Physical examination revealed a pale, sick-looking infant with prolonged capillary refill time and cold extremities. He had a depressed anterior fontanel, dry mucus membranes and sunken eyes. Cardiac and respiratory examinations revealed tachypnea, tachycardia, intercostal and subcostal recessions, and no added sounds. Abdominal examination showed a soft non-tender hepatomegaly with a liver span of 7 cm. A provisional diagnosis of septic shock was entertained. Following routine blood samples, the patient received fluid resuscitation and was commenced on intravenous broad-spectrum antibiotics. The results of complete blood count, serum glucose and renal functions were within normal limits. Arterial blood gas showed compensated mild metabolic acidosis. Liver function tests revealed albumin 18 g/L (normal range, 30–50 g/), alanine aminotransferase 40 U/L (normal range, 20–65 U/L), aspartate aminotransferase 65 U/L (12–37 U/L), alkaline phosphatase 3,547 U/L (normal range, 175–476 U/L), total bilirubin 135 μmol/L (normal range, 2–22 μmol/L) and direct bilirubin 61 μmol/L (normal range, 0–5 μmol/L). Coagulation profile showed prothrombin time 44.2 s (normal range, 11.5–16.5 s), activated partial thromboplastin time 84.9 s (normal range, 26–39 s) and international normalized ratio 4.78 (normal range, 0.3–1.3). Serum calcium and phosphorus levels were normal, serum 25-hydroxyvitamin D was low, and a wrist X- ray showed osteopenia; consistent with vitamin D-deficiency rickets. Echocardiography (Figures 1, 2 and 3) revealed situs solitus, levocardia, concentric biventricular hypertrophy, interventricular septal hypertrophy, mild dilated right atrium and a small, muscular ventricular septal defect. The ventricular ejection fraction was 65%. Metabolic liver disease was suspected on the basis of hepatomegaly, metabolic acidosis, elevated liver enzymes and coagulopathy, and an extensive workup was arranged. Ammonia, lactate, acylcarnitine profile, and galactose-1-phosphate uridyl transferase were normal. Serum amino acid analysis showed tyrosine 440 mmol/L (normal value, < 200 mmol/L), methionine 358 mmol/L (normal value < 63 mmol/L), phenylalanine 188 mmol/L (normal value < 180 mmol/L). Urine organic acid analysis revealed elevated succinylacetone, 15.9 mmol/L (normal value < 0.98 mmol/L), which was consistent with a diagnosis of TT1. An FAH gene study confirmed the presence of a homozygous c.1 A > G mutation. The parents were both heterozygotes for the same mutation. The patient received cardiopulmonary support in the form of mechanical ventilation, dopamine, dobutamine and epinephrine infusion. Specific treatment measures for TTI commenced on the third day of admission, including the use of tyrosine-free formula and 2-Nitro-4-trifluoromethylbenzyl 1, 3 cyclohexanedione (NTBC) administered at 1 mg/kg/day in two divided doses. One week later, the patient was self-ventilating in room air, and all inotropes had been discontinued. After a further week, the patient’s liver function tests and coagulation study results had reverted to normal. Echocardiographic changes normalized after 4 weeks.

Case 2 was a male sibling of Case 1, who was born 6 months after the diagnosis of tyrosinemia had been established in his brother. Pregnancy and delivery were uneventful. Serum amino acid and organic acid analyses 4 days after birth confirmed TT1. A DNA study identified the same c.1 A > G homozygous mutation as in his brother. Echocardiography was normal. Tyrosine-free formula and NTBC were commenced on day 7 of life. This infant remained asymptomatic after 9 months of follow-up.

## Discussion

TT1 is a rare metabolic disorder with an estimated incidence of 1 in 100,000–120,000 births worldwide [1]. TT1 is more common in some regions such as Quebec province in Canada [1,10], but there are no epidemiological data regarding the incidence of TT1 in the Middle East, including Saudi Arabia. However, hospital-based case series and reports from the Middle East, together with the high incidence of consanguinity, suggest that TT1 may be more common than generally believed [2,3,11,12].

In this report, we present two siblings affected by TT1; one diagnosed after presenting with a life-threatening condition, and the other who remained asymptomatic after being diagnosed by selective screening. The diagnosis of TTI in both these patients was based on elevated serum tyrosine levels and the detection of succinylacetone in the urine [2]. The diagnosis was confirmed by the identification of a pathogenic mutation in the FAH gene, which has previously been described in patients from Saudi Arabia and other countries [11,12].

Infants with TT1 usually present in the first few months of life with prolonged jaundice, hepatopathy and failure to thrive [1,3]. Our first patient presented with sepsis-like features and hepatic impairment, including elevated liver enzymes and coagulopathy. This highlights the importance of considering acute metabolic disorders such as tyrosinemia and galactosemia in children who present with unexplained sepsis-like symptoms, especially in the presence of hepatic impairment. Liver damage in TT1 is secondary to the accumulation of the intermediate toxic metabolite fumarylacetoacetate, which results in severe impairment of clotting factor synthesis, leading to bleeding diathesis [13,14]. This process is usually triggered by infections or catabolic stress.

The presence of vitamin D-deficiency rickets in this patient was notable. In contrast, TT1 is known to cause rickets secondary to renal tubular defects as a late manifestation of the disease, which was not seen in our 2-month-old infant. Moreover, the primary biochemical findings in renal rickets associated with tyrosinemia are low serum phosphate and increased urinary phosphate excretion, which were also absent in our patient.

Childhood cardiomyopathy (CM) caused by inborn errors of metabolism is rare [4]. However, CM associated with TT1 has been reported in a few studies [6-9], and Arora et al. reported CM in six out of 20 patients with TT1 [6]. In agreement with our findings, Arora et al. reported that cardiomyopathy associated with tyrosinemia was benign and was associated with a favorable outcome and complete resolution without recurrence in the vast majority of cases [6]. Similar to Case 1 in the present study, André et al. reported the successful treatment of severe cardiomyopathy with NTBC in a patient with TT1 [8]. Furthermore, Mohan et al. reported the complete resolution of cardiomyopathy in three patients with TT1 after liver transplantation [9]. However, in contrast with reports indicating a favorable prognosis of TT1-associated cardiomyopathy, data from the PCMR suggest that hypertrophic cardiomyopathy associated with inborn errors of metabolism carries a poorer prognosis [4]. This could be explained by the fact that most patients with inborn errors of metabolism included in the PCMR database had mitochondrial disorders, which are known to carry a poor prognosis, even in the absence of cardiomyopathy [4]. As seen in Case 1, the type of cardiomyopathy associated with TT1 is usually hypertrophic cardiomyopathy, predominantly involving hypertrophy of the interventricular septum [6-9]. It is interesting to note that most cases of TT1-associated cardiomyopathy are reported at the initial presentation of TT1, as in our first patient [6,7]. Moreover, cardiomyopathy is significantly less common in patients who receive initial treatment with NTBC [6]. Accordingly, we suggest that echocardiography should be considered in the initial assessment of new cases of TT1, especially if they appear acutely sick or have an unexplained illness.

The pathogenesis of cardiomyopathy associated with TT1 is poorly understood [6-8]. It may be related to elevated tyrosine levels or to the accumulation of toxic fumarylacetoacetate in the cardiac tissues. This view is supported by the fact that cardiomyopathy resolves in most patients treated with NTBC or liver transplantation [6-8].

It is interesting that the second infant we report here did not have cardiomyopathy during 9 months of follow-up after birth. This could be because this second infant was diagnosed and treated early, before the development of TT1-associated morbidity, including cardiomyopathy. This suggests that cardiomyopathy secondary to TT1 may be an acquired, rather than a congenital condition. However, this assumption could not be confirmed in the first infant, because he did not receive an echocardiographic examination during the asymptomatic neonatal period. The evidence from these two cases indicates that newborn screening for TT1 may prevent morbidity and improve outcome.

## Conclusions

We report two infants with TT1. One presented with hypertrophic cardiomyopathy but responded dramatically to tyrosine-free formula and NTBC. The second infant was diagnosed during the first week of life and remained asymptomatic after 9 months. This report highlights TT1 as a cause of treatable cardiomyopathy, and demonstrates the value of screening for the early diagnosis of TT1 to prevent associated morbidities.

## Consent

Written informed consent was obtained from the parents of the two siblings for publication of this Case Report and any accompanying images. A copy of the written consent is available for review by the Editor-in-Chief of this journal.

## Abbreviations

TT1: Tyrosinemia type 1; NTBC: 2-Nitro-4-trifluoromethylbenzyl 1, 3 cyclohexanedione.

## Competing interests

All authors declare that they have no competing interests.

## Authors’ contributions

SM designed the paper, collected the data, reviewed the literature, drafted and reviewed the manuscript. MAK, NAA, AA, AB, RH and ASA participated in data collection, writing and reviewing the manuscript. All authors read and approved the final manuscript.

## Authors’ information

Sarar Mohamed (SM) is a Fellow of the Royal College of Pediatrics and Child Health. He is a consultant pediatric endocrinologist and Associate Professor of Pediatrics at King Saud University in Saudi Arabia. Mohammed A. Kambal (MAK) is a pediatric resident at King Saud University. Nasir A. Al Jurayyan (NAA) MD is Professor of Pediatrics and a consultant pediatric endocrinologist at King Saud University. Abdulrahman Al-Nemri (AA) MD is Chairman of the Pediatric Department and Associate Professor of Pediatrics. Amir Babiker (AB) is a member of the Royal College of Pediatrics and Child Health, Assistant Professor of Pediatrics, and a consultant pediatric endocrinologist. Rana Hasanato (RH) MD is Assistant Professor of Pathology and a consultant clinical chemist. Abdulla S. Al-Jarallah (ASA) MD is Professor of Pediatrics and a consultant pediatric cardiologist.

## References

[B1] Mitchell GA Grompe M Lambert M Tanguay RM Scriver CR, Beaudet A, Sly WS, Valle D Hypertyrosinemia : the metabolic and molecular bases of inherited disease 2001 5 New York: McGraw Hill 1777 1806

[B2] Rashed MS Al-Ahaidib LY Al-Dirbashi OY Tandem mass spectrometric assay of succinylacetone in urine for diagnosis of hepatorenaltyrosinemia Anal Biochem 2005 339 310 317 10.1016/j.ab.2005.01.014 15797572

[B3] Rashad M Nassar C Tyrosinemia type 1: a case report Sudan J Pediatr 2011 11 1 64 67 PMC4949785 27493308

[B4] Wilkinson J Sleeper L Alvarez J Bublik N Lipshultz S The pediatric cardiomyopathy registry: 1995–2007 ProgPediatrCardiol 2008 25 1 31 36 10.1016/j.ppedcard.2007.11.006 PMC2408445 19343086

[B5] Nugent AW Daubeney PEF Chondros P Carlin JB Cheung M Wilkinson LC Davis AM Kahler SG Chow CW Wilkinson JL Weintraub RG National Australian childhood cardiomyopathy study.: the epidemiology of childhood cardiomyopathy in Australia N Engl J Med 2003 348 17 1639 1646 10.1056/NEJMoa021737 12711738

[B6] Arora N Staper O Wright J Kelly D Mckiernan P Cardiomyopath in tyrosinemia type 1 is common but usually benign J Inherit Metab Dis 2006 29 54 57 10.1007/s10545-006-0203-5 16601868

[B7] Lindblad B Fallstrom SP Hoyer S Nordborg C Solymar L Velander H Cardiomyopathy in fumarylscetoacetase deficiency (hereditary tyrosinemia): a new feature of the disease J Inherit Metab Dis 1987 10 2 319 322

[B8] Andre’ N Roquelaure B Jubin V Ovaert C Successful treatment of severe cardiomyopathy with NTBC in a child with tyrosinemia type 1 J Inherit Metab Dis 2005 28 1 103 106 10.1007/s10545-005-5085-4 15702412

[B9] Mohan N McKiernan P Preece MA Green A Buckels J Mayer AD Kelly DA Indications and outcome of liver transpalntaion in tyrosinemia type 1 Eur J Pediatr 1999 158 2 249 254 10603099 10.1007/pl00014321

[B10] De Braekeleer M Larochelle J Genetic epidemiology of hereditary tyrosinemia in Quebec and in Saguenary-Lac-St-Jean Am J Hum Genet 1990 47 302 307 2378355 PMC1683702

[B11] Imtiaz F Rashed M Al Mubarak B Allam R El-Karaksy H Al-Hassnan Z Al-Owain M Al-Zaidan H Rahbeeni Z Qari A Meyer BF Al-Sayed M Identification of mutation causing hereditary TyrosinemiaType 1 in patients of Middle Eastern origin Mol Genet Metab 2011 104 4 688 690 10.1016/j.ymgme.2011.06.019 21764616

[B12] Bergman AJ van den Berg IE Brink W Poll-The BT Ploos van Amstel JK Berger R Spectrum of mutations in the fumarylacetoacetatehydrolase gene of tyrosinemia type 1 patients in Northwestern Europe and Mediterranean countries Hum Mutat 1998 12 19 26 10.1002/(SICI)1098-1004(1998)12:1<19::AID-HUMU3>3.0.CO;2-3 9633815

[B13] Croffie JM Gupta SK Chong SK Fitzgerald JF Tyrosinemia type 1 should be suspected in infants with severe coagulopathy even in the absence of other signs of liver failure Pediatrics 1999 103 3 675 678 10.1542/peds.103.3.675 10049978

[B14] Georgouli H Schulpis KH Michelakaki H Kaltsa M Sdogou T Kossiva L Persistent coagulopathy during Escherichia coli sepsis in a previously healthy infant revealed undiagnosed tyrosinaemia type 1 BMJ Case Rep 2010 2010 bcr0720103150 doi:10.1136/bcr.07.2010.3150. 10.1136/bcr.07.2010.3150 22802474 PMC3029516

